# Low-Temperature Sintering and Microwave Dielectric Properties of Cu*_x_*Zn_1−*x*_Ti_0.2_Zr_0.8_Nb_2_O_8_ Ceramics with the Aid of LiF

**DOI:** 10.3390/ma17246251

**Published:** 2024-12-20

**Authors:** Xing-Hua Ma, Qi Qu, Haitao Wu, Zhenlu Zhang, Xingyi Ma

**Affiliations:** 1 School of Mechanical & Automotive Engineering, Qingdao University of Technology, Qingdao 266520, China; 2 School of Environmental and Material Engineering, Yantai University, Yantai 264005, China; 3 School of Science, Beijing University of Posts and Telecommunications, Beijing 100876, China; 4 School of Science, Harbin Institute of Technology, Shenzhen 518055, China

**Keywords:** Cu*_x_*Zn_1−x_Ti_0.2_Zr_0.8_Nb_2_O_8_ ceramics, LiF, LTCC, microwave dielectric properties

## Abstract

*M*^2+^*N*^4+^Nb_2_O_8_-type ceramics (where *M* = Mg, Ca, Mn, Co, Ni, Zn and *N* = Ti, Zr) are essential for satellite communication and mobile base stations due to their medium relative permittivity (*ε*_r_) and high quality factor (*Q* × *f*). Although ZnTi_0.2_Zr_0.8_Nb_2_O_8_ ceramic exhibits impressive microwave dielectric properties, including an *ε_r_* of 29.75, a *Q* × *f* of 107,303 GHz, and a *τ_f_* of −24.41 ppm/°C, its sintering temperature of 1150 °C remains a significant barrier for integration into low-temperature co-fired ceramic (LTCC) technologies. To overcome this limitation, a strategy involving the partial substitution of Zn^2+^ with Cu^2+^ and the addition of LiF as a sintering aid was devised for ZnTi_0.2_Zr_0.8_Nb_2_O_8_. The dual impact of Cu^2+^ partial substitution and LiF as a sintering enhancer facilitated the successful sintering of Cu_0.3_Zn_0.7_Ti_0.2_Zr_0.8_Nb_2_O_8_ ceramics at a reduced temperature of 950 °C using the conventional solid-state reaction method. These ceramics exhibited excellent microwave dielectric properties. Notably, Cu_0.3_Zn_0.7_Ti_0.2_Zr_0.8_Nb_2_O_8_ ceramic with 40 mol% LiF addition demonstrated optimal microwave dielectric properties without any reaction with a silver electrode at a sintering temperature of 950 °C, yielding *ε_r_* = 32, *Q* × *f* = 45,543 GHz, and *τ_f_* = −43.5 ppm/°C.

## 1. Introduction

With the accelerated advancement of 5G and forthcoming 6G technologies, millimeter-wave devices have emerged as a focal area of research on a global scale, attributed to their exceptional ultra-high-speed transmission rates and low latency in communication processes. Microwave dielectric ceramics, distinguished by their superior performance characteristics within the millimeter-wave frequency range, exhibit substantial practical utility across a multitude of communication systems, including radar systems, base station infrastructures, GPS navigational aids, and satellite communications [1,2,3,4,5,6]. In accordance with the specific requirements of practical applications, microwave dielectric materials must possess the following critical attributes: they must exhibit appropriate dielectric constants (*ε_r_*) to facilitate the minimization of device dimensions or the mitigation of signal propagation delays, possess high quality factors (*Q × f*) to effectively curtail signal attenuation, and demonstrate a near-zero temperature coefficient of resonant frequency (*τ_f_*) to enhance the reliability and stability of device applications [7,8,9,10].

Low-temperature co-fired ceramic (LTCC) technology, as a form of multi-layer ceramic manufacturing, has emerged as the preeminent approach in the realm of passive integration. This technology is favored for its ability to embed passive components within the ceramic substrate, thereby delivering exceptional radio frequency (RF) performance while enabling device miniaturization [11,12]. The utilization of silver, characterized by its low melting point of approximately 960 °C, as the metal electrode in compact multi-layer ceramic structures represents a prevalent and efficacious choice, given its cost-effectiveness and superior electrical conductivity. Consequently, to ensure the thermal stability and integrity of silver electrodes during the sintering process, the sintering temperature of LTCC materials is typically controlled to be below 960 °C, with a common practice being to limit it to ≤950 °C. This approach ensures that the silver electrodes do not undergo premature melting or degradation, thereby preserving the overall performance and reliability of the LTCC-based devices.

In recent years, the utilization of microwave dielectric ceramics with medium dielectric constants has become increasingly prevalent in satellite communications, mobile base stations, and various other applications. Among these, the *M*^2+^*N*^4+^Nb_2_O_8_ system (where *M* = Mg, Ca, Mn, Co, Ni, Zn, and *N* = Ti, Zr) has emerged as a highly promising ceramic material, attributed to its exceptional dielectric characteristics. For instance, Wu et al. reported ZnZrNb_2_O_8_ ceramics sintered at 1325 °C for 6 h, exhibiting microwave dielectric properties with *ε_r_* = 26.55, *Q × f* = 68,300 GHz, and *τ_f_* = −32.92 ppm/°C [13]. Subsequently, Xiang et al. substituted Zr^4+^ ions with smaller Ti^4+^ ions, resulting in ZnZr_0.8_Ti_0.2_Nb_2_O_8_ ceramics sintered at 1150 °C with even more outstanding properties: *ε_r_* = 29.75, *Q × f* = 107,303 GHz, and *τ_f_* = −24.41 ppm/°C [14]. However, the sintering temperatures of these reported *M*^2+^*N*^4+^Nb_2_O_8_ ceramics remain excessively high for application in the realm of low-temperature co-fired ceramic (LTCC) technology. Therefore, there is an urgent need to reduce their sintering temperatures to below 960 °C, thereby fulfilling the primary requirement for LTCC compatibility. In our previous studies, we emphasized the beneficial effects of strategically removing trace amounts of high-melting-point precursor materials as an effective means to lower the sintering temperature of microwave dielectric ceramics [12]. Consequently, it is plausible that partially replacing high-melting-point materials with low-melting-point materials may also contribute to reducing the sintering temperature of ceramics. Given that the melting point of CuO is significantly lower than that of ZnO, and the ionic radius of Cu^2+^ (0.73 Å) is comparable to that of Zn^2+^ (0.74 Å), with a difference of less than 15%, Cu^2+^ substitution emerges as a viable approach to achieve low-temperature sintering. Furthermore, low-melting-point compounds have frequently been employed as potent sintering aids, significantly decreasing the sintering temperature of microwave dielectric ceramics to below 960 °C [12,15,16,17,18]. These additive oxides not only melt during the sintering process but also interact with the constituent elements of the matrix ceramics, thereby facilitating ceramic densification through the mechanism of liquid-phase sintering [15]. Among the various sintering aids, LiF, with a melting point of 845 °C, is regarded as one of the most effective and economical additives for lowering the sintering temperature of ceramics [16,17,18]. For example, the sintering temperature of Li_2_Mg_0.2_ZrO_3.2_ ceramics, which exhibit the optimal microwave dielectric properties, can be reduced from 1350 °C to 950 °C with the addition of 2 wt.% LiF [17]. Similarly, the introduction of 8 wt.% LiF lowered the sintering temperature of Li_3_Mg_3_NbO_7_ ceramics from 1200 °C to 900 °C [18].

In this study, a series of novel Cu*_x_*Zn_1−x_Ti_0.2_Zr_0.8_Nb_2_O_8_ ceramics, with compositions ranging from *x* = 0.0 to *x* = 0.4, were synthesized using the conventional solid-state reaction method. Specifically, Cu_0.3_Zn_0.7_Ti_0.2_Zr_0.8_Nb_2_O_8_ (i.e., *x* = 0.3) was selected for further investigation due to its relatively optimal performance within this series, and varying amounts of LiF were introduced into this composition to strive for a sintering temperature below 960 °C. A meticulous examination was then conducted to explore in depth the microstructural alterations and microwave dielectric properties of the LiF-doped Cu_0.3_Zn_0.7_Ti_0.2_Zr_0.8_Nb_2_O_8_ ceramics. Additionally, the chemical compatibility of the ceramics with a silver electrode was evaluated. The findings of this work underscore a promising approach for the development of *M*^2+^*N*^4+^Nb_2_O_8_-type ceramics that not only exhibit low sintering temperatures but also possess exceptional microwave dielectric characteristics.

## 2. Materials and Methods

The Cu*_x_*Zn_1−x_Ti_0.2_Zr_0.8_Nb_2_O_8_ ceramics (0.0 ≤ *x* ≤ 0.4) were synthesized using analytical-grade ZnO (Makclin Biochemical Technology, Shanghai, China), CuO, ZrO_2_, TiO_2_, and Nb_2_O_5_ (Aladdin Biochemical Technology, Shanghai, China) via the conventional solid-state reaction method. This method includes ball milling and subsequent high-temperature treatment (calcination and sintering). Ball milling allows for the uniform mixing of different types of powders or compounds and high-temperature treatment provides sufficient energy to overcome reaction barriers and enable the reaction to proceed. In comparison to other chemical synthesis methods, despite its time-consuming nature and high energy consumption, the solid-state reaction method offers a multitude of advantages, including simplicity in operation, ease of control, excellent crystallinity of the resulting ceramics, strong customizability, and the capability to easily achieve large-scale production [19]. In the context of the present research, a typical experimental procedure is detailed as outlined below.

The raw powders, weighing a total of 28 g and proportioned according to the stoichiometric requirements, were ball-milled in a Teflon jar containing zirconia balls of various sizes (specifically, 2 pieces of 20 mm, 10 pieces of 15 mm, 40 pieces of 10 mm, 120 g of 5 mm, and 80 g of 3 mm) and 33 milliliters of ethanol. The milling process was conducted on a horizontal ball mill operating at a speed of 25 rpm for a duration of 24 h. The slurry was subsequently dried at 85 °C for 4 h followed by calcination at 900 °C for 3 h. The calcined powders underwent a secondary milling process, employing the same parameters as the initial stage, for an additional 24 h in order to achieve a finer powder consistency. Subsequently, they were dried at 85 °C for another 4 h and sifted through a 40-mesh sieve. Finally, after grinding with 5 wt.% PVA (as the organic binder) in an agate mortar and sifting through a 200-mesh sieve, the resulting powders were pressed into cylindrical disks of 10 mm in diameter and 5 mm in height under a pressure of 100 MPa. To extract the organic binder, the green pellets were firstly heated at 600 °C for 2 h, followed by sintering at a temperature range of 1050–1300 °C for 4 h. To further decrease the sintering temperature, various amounts of LiF (0.0 mol–50.0 mol%, 99.9%, Makclin Biochemical Technology, Shanghai, China) were added during the second ball milling, and a similar procedure to that described above was performed, except the green pellets were sintered at 950 °C for 4 h.

The relative densities of sintered specimens were determined using the Archimedes method, employing DI water as the buoyancy medium. The phase composition and purity were analyzed using an X-ray diffractometer (XRD; Rigaku SmartLab SE, Tokyo, Japan) equipped with Cu Kα radiation. The XRD testing parameters were precisely defined: the 2θ range was set from 10° to 90°, with a step size of 0.01° and a scanning speed of 2° per minute. Additionally, the operating voltage and current were adjusted to 40 kV and 40 mA, respectively. The crystal structure of all compositions was further analyzed by using the TOPAS-Academic Rietveld refinement method and Raman spectrometer (LabRam HR Evolution, HORIBA Scientific, Kyoto, Japan) with an argon laser (532 nm). The microstructure of the polished and thermally etched (conducted at 100 °C below the optimum sintering temperature for 1 h) samples was observed using scanning electron microscopy (SEM; Zeiss Sigma 300, Oberkochen, Germany), and compositional analysis was performed using an energy-dispersive spectroscope (EDS; Oxford UltimMax40, UK) attached to the SEM. The average grain sizes were estimated using ImageJ software (v1.8.0). The valence states of elements were analyzed through X-ray photoelectron spectroscopy (XPS; Thermo Scientific K-Alpha, Waltham, MA, USA). The microwave dielectric properties were evaluated with a network analyzer (3656D, Ceyear, Qingdao, China) connected with a temperature chamber, as suggested by Hakki-Coleman and Courtney [20,21]. Permittivity (*ε_r_*) and quality factors (*Q × f*) were determined in TE_01δ_ mode. Temperature coefficients of the resonant frequencies (*τ_f_*) were measured in the temperature range of 25–85 °C and then calculated using the following equation [22]:

(1) $$\tau_{f}=\frac{f_{2}-f_{1}}{f_{1}\left(T_{2}-T_{1}\right)}$$

where *f*_1_ and *f*_2_ represent the resonant frequency at *T*_1_ (25 °C) and *T*_2_ (85 °C), respectively.

## 3. Results and Discussion

**Figure 1:**
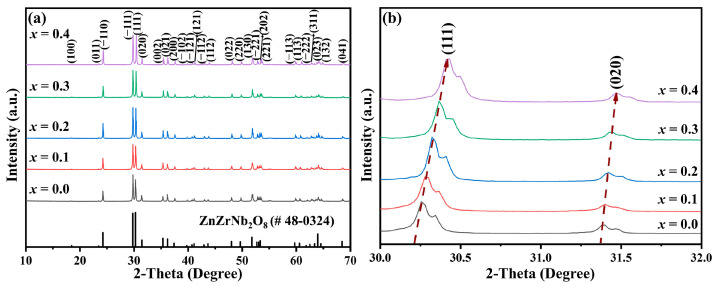
(**a**) XRD patterns of Cu*_x_*Zn_1−x_Ti_0.2_Zr_0.8_Nb_2_O_8_ ceramics (0.0 ≤ *x* ≤ 0.4); (**b**) enlarged XRD patterns in the range of 30°–32°.

The XRD patterns of Cu*_x_*Zn_1−x_Ti_0.2_Zr_0.8_Nb_2_O_8_ ceramics (0.0 ≤ *x* ≤ 0.4) sintered at 1050 °C–1300 °C for 4 h are shown in Figure 1a. With the increase in *x* values, all patterns could be indexed to the ZnZrNb_2_O_8_ diffraction profile with JCPDS card #48-0324 [14], and no obvious secondary phase appeared. The second phase, including CuZrNb_2_O_8_ and CuTiNb_2_O_8_, was not detected, indicating that Cu^2+^ entered the ZnTi_0.2_Zr_0.8_Nb_2_O_8_ lattice and formed a solid solution. In addition, it was observed that the (111) and (020) peaks tended towards a higher diffraction angle, as shown in Figure 1b, indicating a gradual decrease in the unit cell volume. This is possibly due to the smaller ionic radius of Cu^2+^ (0.73 Å) than that of Zn^2+^ (0.74 Å) [23].

**Figure 2:**
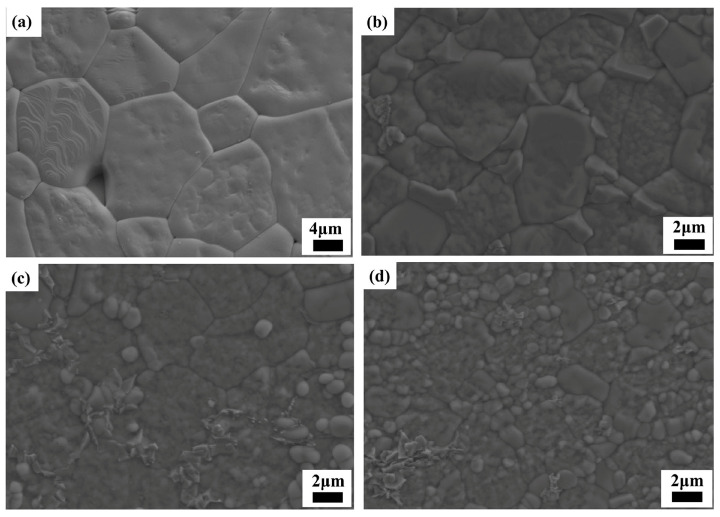
SEM images of Cu*_x_*Zn_1−x_Ti_0.2_Zr_0.8_Nb_2_O_8_ (0.0 ≤ *x* ≤ 0.4) specimens: (**a**) *x* = 0.0; (**b**) *x* = 0.2; (**c**) *x* = 0.3; (**d**) *x* = 0.4.

Figure 2 shows the SEM images of the Cu*_x_*Zn_1−x_Ti_0.2_Zr_0.8_Nb_2_O_8_ (0.0 ≤ *x* ≤ 0.4) ceramics after thermal etching treatment. The specimen with *x* = 0.0 exhibits a microstructure consisting of large grains with an average grain size of approximately (12.2 ± 4.1) μm (Figure 2a). At *x* = 0.2, the average grain size decreased to around (5.9 ± 1.3) μm (Figure 2b), probably because of the appearance of a secondary phase along the grain boundaries, which inhibited the grain growth. As *x* values further increased, the grain size decreased significantly and an average grain size of (3.6 ± 0.9) μm could be found for *x* = 0.4 (Figure 2d). To recognize the secondary phase along the grain boundaries, EDS mapping analysis was conducted on the specimen with *x* = 0.3 (Figure S1 of the Supplementary Material), and the precipitated phase formed along the grain boundaries could be recognized as a Cu-rich phase. Because CuO is usually employed as the sintering aid for the ceramic densification at lower temperature through the mechanism of liquid-assisted sintering [24,25,26], and the current specimens were sintered at a high temperature of 1050–1300 °C, a liquid phase abundant in Cu readily formed along the grain boundaries.

**Figure 3:**
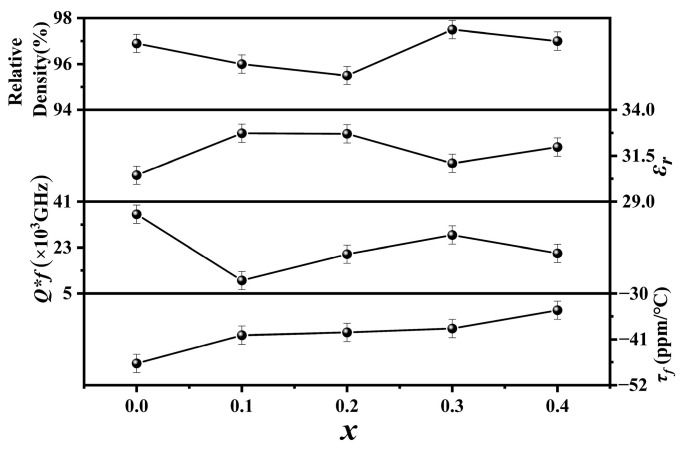
Relative densities and microwave performance of Cu*_x_*Zn_1−x_Ti_0.2_Zr_0.8_Nb_2_O_8_ (0.0 ≤ *x* ≤ 0.4) ceramics.

Figure 3 shows the relative densities and microwave dielectric properties of Cu*_x_*Zn_1−x_Ti_0.2_Zr_0.8_Nb_2_O_8_ ceramics (0.0 ≤ *x* ≤ 0.4). The relative densities of all the specimens exhibited high values (≥95%), indicating that they were well sintered under the current conditions. A maximum value of ~97.5% was achieved at *x* = 0.3. As the *x* values increased, the *ε_r_* values fluctuated within a narrow range of 30–33. As reported by Zhang et al., the substitution of Cu^2+^ will deteriorate the microwave dielectric properties [24]. Therefore, compared to the specimen with *x* = 0.0 (i.e., ZnTi_0.2_Zr_0.8_Nb_2_O_8_), the *Q × f* values decreased obviously. Nevertheless, the *Q × f* values were still higher than 5000 GHz, especially the specimen with *x* = 0.3, which exhibited its highest *Q × f* value of around 28,000 GHz when 0.1 ≤ *x* ≤ 0.4, probably due to its highest relative density. The *τ_f_* values positively shifted from −47 ppm/°C to −34 ppm/°C, probably due to the effect of Cu^2+^ substitution.

**Figure 4:**
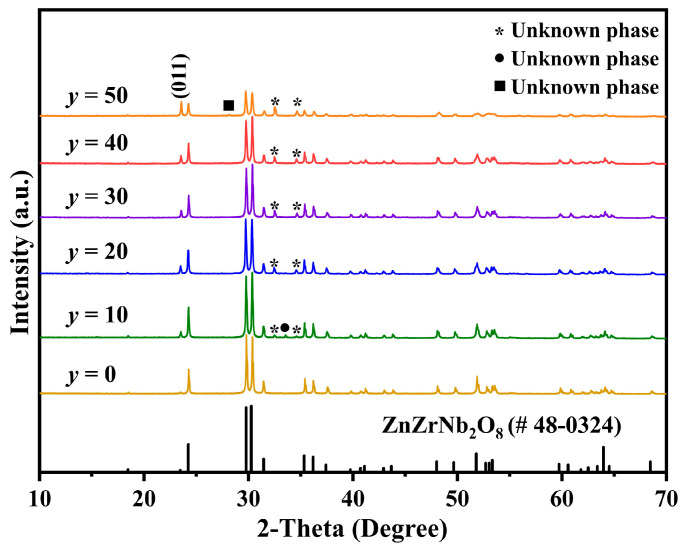
XRD patterns of Cu_0.3_Zn_0.7_Ti_0.2_Zr_0.8_Nb_2_O_8_ supplemented with *y* mol% LiF (0 ≤ *y* ≤ 50).

Because the highest *Q × f* value was achieved at *x* = 0.3, in the next step, LiF-added Cu_0.3_Zn_0.7_Ti_0.2_Zr_0.8_Nb_2_O_8_ ceramics were synthesized, and their potential for LTCC applications was further evaluated. Figure 4 shows the XRD patterns of Cu_0.3_Zn_0.7_Ti_0.2_Zr_0.8_Nb_2_O_8_ ceramics supplemented with *y* mol% LiF (10 ≤ *y* ≤ 50) and without LiF (i.e., *y* = 0) sintered at 950 °C and 1100 °C for 4 h, respectively. After adding the various amounts of LiF, the majority diffraction peaks could still be indexed well to the ZnZrNb_2_O_8_ diffraction profile with JCPDS card #48-0324. However, an unidentified phase, denoted by asterisks, emerged consistently within the range of 32° to 35° in all specimens containing LiF additions. Notably, this unknown phase persisted even after sintering at approximately 1100 °C. Furthermore, its peak intensity augmented as the *y* values increased, suggesting a direct correlation with the addition of LiF. Additionally, the intensity of the (011) peak was enhanced with increasing LiF content, indicating that the diffraction peak of a substance associated with LiF also manifests at this specific position. Moreover, an impurity phase, highlighted by a solid circle, was detected in the specimen with *y* = 10, while another impurity phase, denoted by a solid square, appeared in the specimen with *y* = 50. Given their absence in other X-ray diffraction (XRD) patterns, these impurities are likely attributed to other elements rather than LiF. Furthermore, the diffraction peak intensity of the specimen with *y* = 50 exhibited a marked decrease, indicating a significant increase in the formation of the liquid phase within the material. This observation is supported by the substantial reduction in relative density observed in subsequent stages, corroborating the conclusion regarding the increased liquid phase formation.

**Figure 5:**
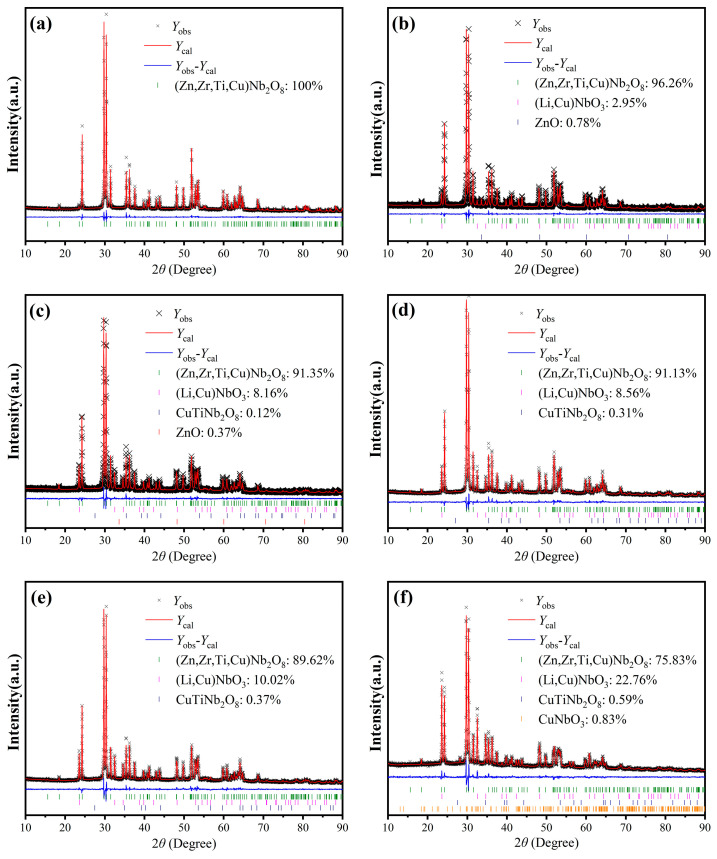
Rietveld refinement XRD profiles of Cu_0.3_Zn_0.7_Ti_0.2_Zr_0.8_Nb_2_O_8_ supplemented with *y* mol% LiF (0 ≤ *y* ≤ 50): (**a**) *y* = 0; (**b**) *y* = 10; (**c**) *y* = 20; (**d**) *y* = 30; (**e**) *y* = 40; (**f**) *y* = 50.

To further investigate the variations in crystal structure and the unknown phases upon LiF addition, Rietveld refinements were conducted. The corresponding XRD refinement patterns are shown in Figure 5, where (a)–(f) stand for the representative results for samples with *y* = 0, 10, 20, 30, 40, and 50, respectively. The refined structural parameters for Cu_0.3_Zn_0.7_Ti_0.2_Zr_0.8_Nb_2_O_8_ ceramics with 0 mol% and 40 mol% LiF are selectively listed in Table S1 of the Supplementary Materials. As revealed by the results, the (Zn, Zr, Ti, Cu)Nb_2_O_8_ phase dominates in all the specimens. However, during the LiF doping process, the formation of the (Li, Cu)NbO_3_ secondary phase was observed in all the specimens, indicating that the unknown phase marked by the asterisks in Figure 4 was confirmed to be (Li, Cu)NbO_3_. Besides the (Li, Cu)NbO_3_ phase, small amounts of ZnO impurities with 0.78 wt.% and 0.37 wt.% were also recognized in the specimen with *y* = 10 (it should correspond to the peak marked by a solid circle in Figure 4) and *y* = 20, respectively. Due to its relatively low content, ZnO was not visible in the XRD pattern for *y* = 20. As the amount of LiF doping increased, the proportion of the (Li, Cu)NbO_3_ phase showed a positive correlation with the doping level *y* (this phenomenon can also be clearly demonstrated by the increased intensity of the (011) peak, as well as the peaks denoted with asterisks), while the content of the (Zn, Cu, Ti, Zr)Nb_2_O_8_ phase decreased correspondingly. Additionally, CuTiNb_2_O_8_ emerged and increased progressively as *y* varied from 20 to 50 (the impurity phase denoted by a solid square in Figure 4 just corresponds to it), probably because the LiF with a lower melting point further promoted the formation of Cu-related liquid phase. This can be proved by the specimen with *y* = 50, where a small amount of CuNbO_3_ was observed, indicating that Cu^2+^ was reduced to Cu^+^ possibly due to the reduction in divalent Cu ions under oxygen-deficient conditions during sintering at 950 °C. To further clarify this phenomenon, XPS spectra of the specimen with *y* = 50 were measured and presented in Figure S2. The survey spectrum depicted in Figure S2a clearly exhibits peaks corresponding to Li 1s, Zr 3d, Nb 3d, Ti 2p, O 1s, F 1s, Cu 2p, and Zn 2p, providing additional evidence for the presence of Li and F, despite their non-detection by the EDS, as noted in the subsequent section. Furthermore, the peak-fitting curves of both the Cu 2p_3/2_ and Cu 2p_1/2_ peaks shown in Figure S2b can be split into two peaks by Gaussian–Lorentzian curve fitting, respectively, indicating the co-existence of Cu^+^ and Cu^2+^ in this specimen [27].

**Figure 6:**
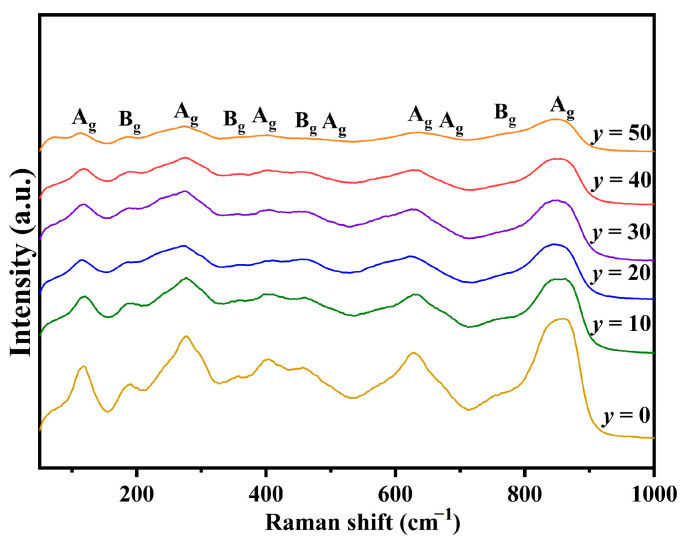
Raman spectra of Cu_0.3_Zn_0.7_Ti_0.2_Zr_0.8_Nb_2_O_8_ supplemented with *y* mol% LiF (0 ≤ *y* ≤ 50).

In addition to XRD, Raman scattering is a powerful tool for investigating lattice vibration, which significantly impacts both the polarization and structural attributes of ceramics. Furthermore, the intrinsic loss in microwave dielectric ceramics is predominantly determined by the anharmonicity and damping of the lattice phonon [28]. Consequently, Raman spectroscopy was employed to conduct a more in-depth exploration of the crystal structures and vibrational modes. Figure 6 shows the corresponding Raman spectra of *y* mol% LiF-added Cu_0.3_Zn_0.7_Ti_0.2_Zr_0.8_Nb_2_O_8_ ceramics (0 ≤ *y* ≤ 50). As indicated by the XRD patterns, all the ceramic specimens with different amounts of added LiF mainly have a monoclinic structure, as exhibited by ZnZrNb_2_O_8_. According to group theory calculations, ZnZrNb_2_O_8_ ceramics possess a monoclinic columbite structure with space group P2/c (No. 013) and point group C2h (2/m), encompassing 39 vibrational modes (Γ = 9*A*_g_ + 8*A*_u_ + 12*B*_g_ + 10*B*_u_), including Raman-active modes (9*A*_g_ + 12*B*_g_) and infrared-active modes (8*A*_u_ + 10*B*_u_) [13,14]. However, the actual number of observed Raman peaks is fewer than theoretically predicted, which may be attributed to the broadening effect or overlap of weak vibrational peaks [14]. Based on previous reports [14,29,30,31], the high-intensity modes at around 845 cm^−1^ and 880 cm^−1^ correspond to symmetric Nb-O bond vibrations that can be found due to a high covalent bond, while the asymmetric Nb-O bond vibrations appear at 770 cm^−1^. For the lower Raman shift, the modes at 635 cm^−1^ and 674 cm^−1^ are related to the asymmetric vibrations of the Nb-O-Nb bond, and other vibrations correspond to symmetric Nb-O-Nb vibrations. Two modes at 355 cm^−1^ and 466 cm^−1^ are assigned to O-Zr-O bending, whereas the 186 cm^−1^ and 408 cm^−1^ modes originate from the stretching vibration of the Zr-O bond. Notably, the incorporation of LiF significantly affects the intensity of each vibrational peak, which may be attributed to changes in the relative concentration within the material system, thereby modulating the vibrational characteristics.

**Figure 7:**
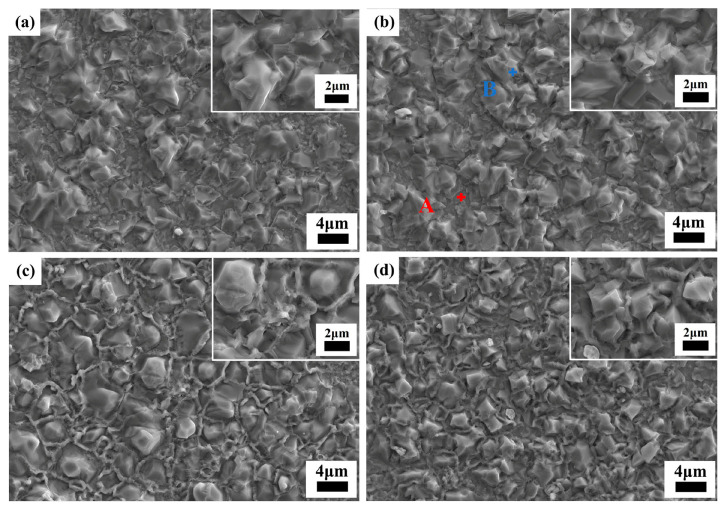
SEM images of Cu_0.3_Zn_0.7_Ti_0.2_Zr_0.8_Nb_2_O_8_ supplemented with *y* mol% LiF (0 ≤ *y* ≤ 50): (**a**) *y* = 10; (**b**) *y* = 20 (Point A was taken from the matrix, while B was taken from precipitated grain); (**c**) *y* = 40; (**d**) *y* = 50.

Figure 7 shows the SEM images of the thermally etched interior surface of *y* mol% LiF-added Cu_0.3_Zn_0.7_Ti_0.2_Zr_0.8_Nb_2_O_8_ ceramics (10 ≤ *y* ≤ 50, the SEM image with *y* = 0 is shown in Figure 2c). In all the images, there is a notable absence of any visible holes, conclusively suggesting that densification sintering was successfully achieved for all the specimens under their respective conditions. Moreover, the morphology of the specimens underwent significant changes after doping with LiF. The morphology of the grains in the specimen with *y* = 10 is uneven (Figure 7a), with an average grain size of approximately (6.0 ± 1.4) μm. Some precipitated grains can be found on the matrix. At *y* = 20 (Figure 7b), the average grain size decreased to approximately (4.8 ± 1.2) μm, accompanied by blurred grain boundaries. Additionally, the surface of the matrix ceramic exhibited a notable presence of precipitated grains. Subsequent analysis via EDS (Figure S3 of the Supplementary Materials) reveals that the precipitated grains (marked as point B in Figure 7b) are mainly composed of Cu-rich phase, which is consistent with the analysis of XRD refinement. The composition of the matrix (marked as point A in Figure 7b) exhibits a Cu-deficient Cu_0.3_Zn_0.7_Ti_0.2_Zr_0.8_Nb_2_O_8_ phase, which is probably due to some of the Cu diffusing into the precipitated grains. When *y* = 40 (Figure 7c), the average grain size decreased even further to approximately (4.6 ± 0.7) μm, with the grain morphology becoming more uniform and the grain boundary clarity increasing, and the composition of the precipitated phase is similar to that of *y* = 20. By *y* = 50 (Figure 7d), the grain size significantly decreased to approximately (3.0 ± 0.4) μm. After doping with LiF, grain refinement was observed, indicating that LiF plays a regulatory role in the grain growth process of Cu_0.3_Zn_0.7_Ti_0.2_Zr_0.8_Nb_2_O_8_ ceramics. The reduction in average grain size may be attributed to the potential decrease in the optimal sintering temperature and the inhibition of grain growth by the higher surface energy associated with increasing sintering aid (i.e., LiF) doping levels [32].

**Figure 8:**
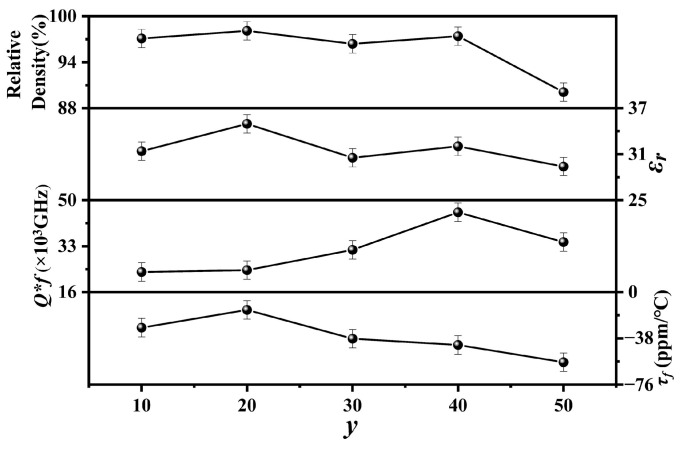
Relative densities and microwave performance of Cu_0.3_Zn_0.7_Ti_0.2_Zr_0.8_Nb_2_O_8_ supplemented with *y* mol% LiF (10 ≤ *y* ≤ 50).

Figure 8 shows the relative densities, *ε_r_*, *Q × f*, and *τ_f_* values of the *y* mol% LiF (10 ≤ *y* ≤ 50)-added Cu_0.3_Zn_0.7_Ti_0.2_Zr_0.8_Nb_2_O_8_ ceramics sintered at 950 °C for 4 h. The relative densities of the specimens with 10 ≤ *y* ≤ 40 exhibited high values (≥95%), indicating that they were well sintered under the current conditions. However, when *y* = 50, its relative density decreased significantly to around 90%, probably due to the generation of an excessive liquid phase caused by the LiF. The trend of *ε_r_* is similar to that of relative density, both of which first increased and then decreased with the addition of LiF, followed by another rise and subsequent fall. As the quantity of LiF increased, the *Q × f* values discernibly followed an upward trend, culminating in a peak value of approximately 45,543 GHz at *y* = 40. However, when *y* exceeded 40, the *Q × f* value decreased to 34,536 GHz at *y* = 50, probably because of the sharply decreased relative density. For mixtures, *τ_f_* is mainly related to the phase composition. For the specimens with low LiF addition, the variation in *τ_f_* values is nearly identical to *ε_r_*. However, when *y* ≥ 30, the deeply negative *τ_f_* of LiF (−117.7 ppm/°C) [33] deteriorates the *τ_f_* of the specimen with *y* = 50 to around −57.7 ppm/°C. Overall, the 40 mol% LiF-added Cu_0.3_Zn_0.7_Ti_0.2_Zr_0.8_Nb_2_O_8_ ceramics sintered at 950 °C for 4 h showed good microwave dielectric properties: *ε_r_* of 32, *Q × f* of 45,543 GHz, and *τ_f_* of −43.5 ppm/°C. Furthermore, when compared to the *M*^2+^*N*^4+^Nb_2_O_8_-type ceramics listed in Table 1, which have been reported in recent years, the Cu_0.3_Zn_0.7_Ti_0.2_Zr_0.8_Nb_2_O_8_ ceramics doped with 40 mol% LiF exhibit an exceptionally outstanding performance in terms of both sintering temperature and microwave dielectric properties.

**Figure 9:**
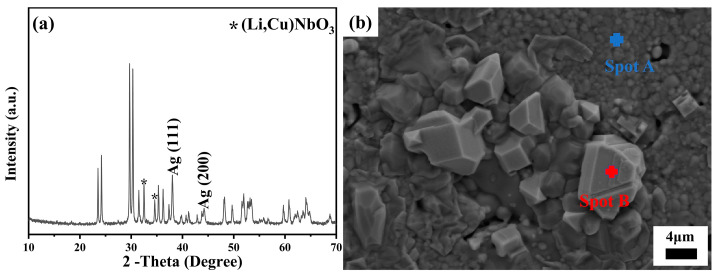
(**a**) XRD pattern and (**b**) SEM image of the 40 mol% LiF-doped Cu_0.3_Zn_0.7_Ti_0.2_Zr_0.8_Nb_2_O_8_ ceramic cofired with 20 wt.% silver powder (Point A was taken from the matrix, while B was taken from Ag powder).

Silver, owing to its exceptional conductivity coupled with a comparatively economical cost, has been ubiquitously employed as an electrode in LTCC devices. Consequently, to ascertain its aptness for LTCC applications, a thorough examination was undertaken to evaluate the chemical compatibility of a 40 mol% LiF-doped Cu_0.3_Zn_0.7_Ti_0.2_Zr_0.8_Nb_2_O_8_ ceramic with silver. To this end, a strategic addition of 20 wt.% silver powder was incorporated into the base powder mixture, which was then subjected to a co-sintering process at a temperature of 950 °C for a duration of 4 h. Figure 9 shows the XRD pattern and SEM image of the as-tested specimen. Remarkably, apart from the distinct diffraction peaks attributed to Ag, specifically identified as Ag (111) and Ag (200), no discernible secondary phase related to Ag addition was detected, unequivocally indicating the absence of any chemical interaction between the ceramic and the introduced silver particles (Figure 9a). This observation was reinforced by the SEM micrograph in Figure 9b, which distinctly showcased a well-defined interface separating the ceramic matrix from the silver particles. Concurrently, the EDS analysis (Figure S4) precisely pinpointed the chemical composition of Spot B as pure Ag, whereas Spot A was characteristic of the grains comprising the 40 mol% LiF-doped Cu_0.3_Zn_0.7_Ti_0.2_Zr_0.8_Nb_2_O_8_ ceramic. These findings unequivocally underscore the excellent chemical stability between the ceramic and the silver electrode at 950 °C, positioning the 40 mol% LiF-doped Cu_0.3_Zn_0.7_Ti_0.2_Zr_0.8_Nb_2_O_8_ ceramic as a highly promising candidate for LTCC applications.

## 4. Conclusions

The *M*^2+^*N*^4+^Nb_2_O_8_ (where *M* = Mg, Ca, Mn, Co, Ni, Zn and *N* = Ti, Zr) ceramics exhibit remarkable microwave dielectric properties. However, their inherently high sintering temperatures pose challenges for their application in low-temperature co-fired ceramic (LTCC) technologies. To address this limitation, a strategic approach involving the partial substitution of Cu^2+^ and the addition of LiF was employed to explore the feasibility of achieving low-temperature sintering. Through a meticulously designed series of orthogonal experiments, it was discovered that Cu_0.3_Zn_0.7_Ti_0.2_Zr_0.8_Nb_2_O_8_ ceramic, characterized by an impressively high quality factor, could be successfully densified at 950 °C with the assistance of LiF. Specifically, the ceramic doped with 40 mol% LiF, when sintered at 950 °C for 4 h, exhibited exceptional microwave dielectric characteristics, including a relative permittivity (*ε_r_*) of 32, a quality factor (*Q × f*) of 45,543 GHz, and a temperature coefficient of resonant frequency (*τ_f_*) of −43.5 ppm/°C. Furthermore, our studies revealed no adverse chemical reactions between silver and the 40 mol% LiF-doped Cu_0.3_Zn_0.7_Ti_0.2_Zr_0.8_Nb_2_O_8_ ceramic, confirming their compatibility. In summary, this work presents an effective strategy to reduce the sintering temperature of *M*^2+^*N*^4+^Nb_2_O_8_ ceramics to an LTCC-compatible level of 950 °C. The 40 mol% LiF-doped Cu_0.3_Zn_0.7_Ti_0.2_Zr_0.8_Nb_2_O_8_ ceramic emerges as a promising candidate for LTCC applications, offering a viable solution to the temperature constraints associated with traditional sintering processes. Additionally, ongoing research is focused on exploring the specific applications of this material.

## Figures and Tables

**Table 1:** The comparison between our reported ceramics with other *M*^2+^*N*^4+^Nb_2_O_8_-type ceramics in terms of sintering temperature and microwave dielectric properties.

Compound	S.T. (℃)	*ε_r_*	*Q × f (*GHz)	*τ_f_* (ppm/°C)	Reference
ZnZrNb_2_O_8_	1325	26.55	68,300	−32.92	[13]
ZnZr_0.8_Ti_0.2_Nb_2_O_8_	1150	29.75	107,303	−24.41	[14]
Zn(Ti_0.6_Zr_0.4_)Nb_2_O_8_	1120	33.43	59,475	−76.54	[34]
CoTiNb_2_O_8_	1000	64.19	16,800	+66.17	[35]
ZnTi_0.97_Ge_0.03_Nb_2_O_8_	1120	35.6	62,700	−58	[36]
ZnTi_0.8_Sn_0.2_Nb_2_O_8_	1120	30.88	43,500	−54.32	[37]
Mn_1.05_Ti_0.9_Zr_0.1_Nb_2_O_8_	1200	32.9	11,427	0	[38]
Zn_0.95_Mn_0.05_TiNb_2_O_8_	1100	35.91	63,672	−68.68	[39]
ZnTi_0.99_Mo_0.01_Nb_2_O_8_	1150	33.91	64,136	−50.57	[40]
(Co_0.35_Zn_0.65_)TiNb_2_O_8_ + 4 wt. %BaCu(B_2_O_5_)	925	36.7	19,880	+14.2	[41]
Cu_0.3_Zn_0.7_Zr_0.8_Ti_0.2_Nb_2_O_8_ + 40 mol% LiF	950	32	45,543	−43.5	This work

## Data Availability

The original contributions presented in this study are included in the article and Supplementary Material. Further inquiries can be directed to the corresponding author.

## Supplementary Materials

The following supporting information can be downloaded at https://www.mdpi.com/article/10.3390/ma17246251/s1, Figure S1: Mapping Spectra of Cu_0.3_Zn_0.7_Ti_0.2_Zr_0.8_Nb_2_O_8_ ceramic; Table S1: Refined crystal structure parameters of Cu_0.3_Zn_0.7_Ti_0.2_Zr_0.8_Nb_2_O_8_ supplemented with *y* mol% LiF and residual factors; Figure S2: XPS spectra of Cu_0.3_Zn_0.7_Ti_0.2_Zr_0.8_Nb_2_O_8_ supplemented with 50 mol% LiF; Figure S3: EDS analysis of Cu_0.3_Zn_0.7_Ti_0.2_Zr_0.8_Nb_2_O_8_ supplemented with 20 mol% LiF; Figure S4: EDS analysis of Cu_0.3_Zn_0.7_Ti_0.2_Zr_0.8_Nb_2_O_8_ supplemented with 40 mol% LiF with Ag cofired.
