# Supplementary material for: Low-Temperature Sintering and Microwave Dielectric Properties of CuxZn1−xTi0.2Zr0.8Nb2O8 Ceramics with the Aid of LiF
Source: Materials (Basel). 2024 Dec 20;17(24):6251. doi: 10.3390/ma17246251 (PMC11678219; doi:10.3390/ma17246251)
Supplement: Supplementary file 1 [file materials-17-06251-s001.zip › materials-3361345-supplementary.pdf]

Supplementary Material

Low-temperature sintering and microwave dielectric properties of $Cu_xZn_{1-x}Ti_{0.2}Zr_{0.8}Nb_2O_8$ ceramics with the Aid of LiF

Xing-Hua Ma[1,*], Qi Qu[1], Haitao Wu[2] , Zhenlu Zhang[3], Xingyi Ma[4]

1. School of Mechanical & Automotive Engineering, Qingdao University of Technology, Qingdao 266520, China;

2. School of Environmental and Material Engineering, Yantai University, Yantai, 264005, China;

3. School of Science, Beijing University of Posts and Telecommunications, Beijing 100876, China;

4. School of Science, Harbin Institute of Technology, Shenzhen, Guangdong 518055, China.

*Corresponding author: maxinghua@qut.edu.cn

1. Mapping Spectra of $Cu_{0.3}Zn_{0.7}Ti_{0.2}Zr_{0.8}Nb_2O_8$ ceramic

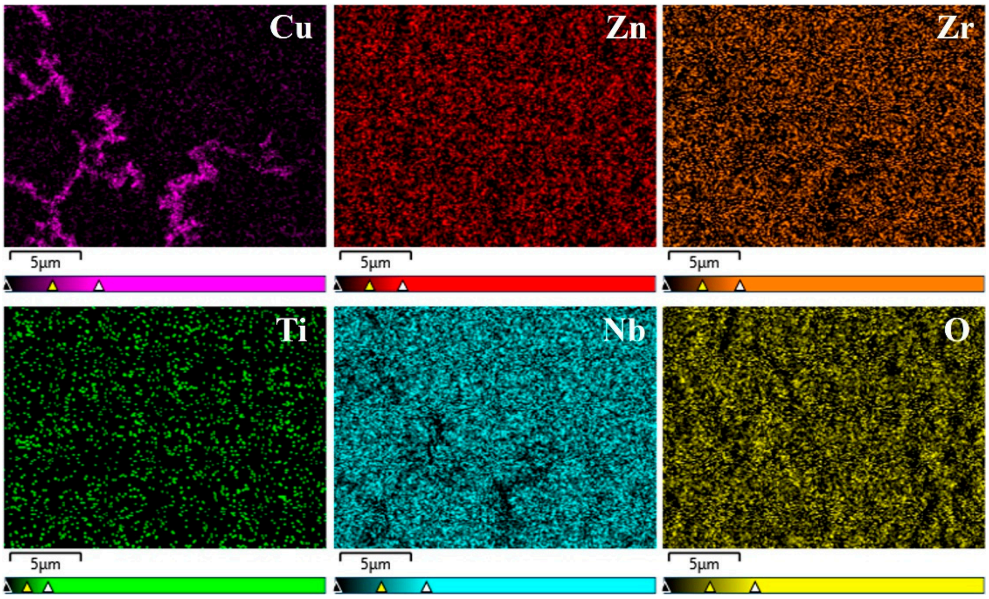

Figure S1. Mapping spectra of $Cu_{0.3}Zn_{0.7}Ti_{0.2}Zr_{0.8}Nb_2O_8$ ceramic

2. Refined crystal structure parameters of $Cu_{0.3}Zn_{0.7}Ti_{0.2}Zr_{0.8}Nb_2O_8$ supplemented with *y* mol% LiF and residual factors

Table S1. Refined crystal structure parameters of $Cu_{0.3}Zn_{0.7}Ti_{0.2}Zr_{0.8}Nb_2O_8$ supplemented with *y* mol% LiF and residual factors (take *y* = 0 and 40 as examples)

y	Phase	Space group	Phase fraction (wt. %)	Lattice parameters (Å)				R factor (%)		
				a (Å)	*b* (Å)	*c* (Å)	*β* (°)	R_p	R_{exp}	GOF
0	(Zn,Zr,Ti,Cu)Nb_2O_8	P12/c1	100	4.77955(13)	5.67712(14)	5.06107(13)	91.48443(99)	5.99	2.03	3.75
40	(Zn,Zr,Ti,Cu)Nb_2O_8	P12/c1	89.62	4.78891(33)	5.67213(39)	5.06567(33)	91.5341(15)	5.29	1.97	3.38
	(Li,Cu)NbO_3	R3cH	10.02	5.17391(42)	5.17391(42)	13.9316(15)				
	$CuTiNb_2O_8$	P42/mnm	0.37	4.5965(16)	4.5965(16)	3.0318(33)				

3. XPS spectra of $Cu_{0.3}Zn_{0.7}Ti_{0.2}Zr_{0.8}Nb_2O_8$ supplemented with 50 mol% LiF

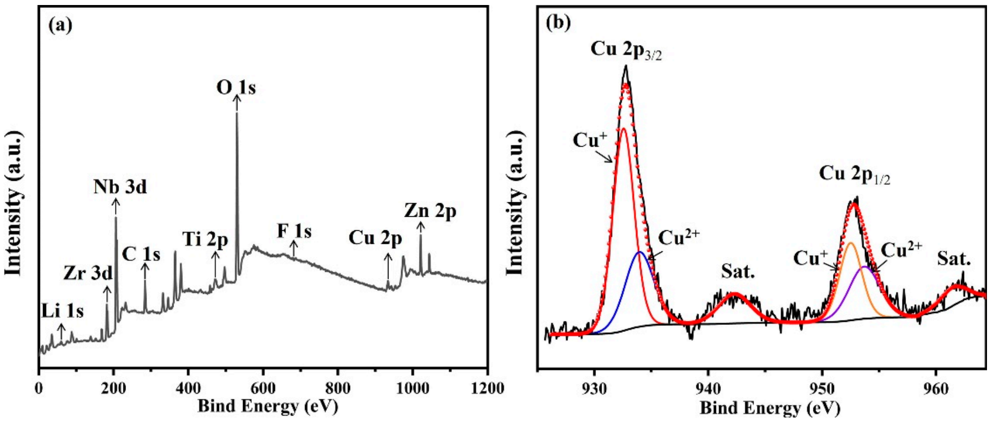

Figure S2. XPS spectra of $Cu_{0.3}Zn_{0.7}Ti_{0.2}Zr_{0.8}Nb_2O_8$ supplemented with 50 mol% LiF (a) the survey spectrum, (b) the detail spectrum of Cu

4. EDS analysis of $Cu_{0.3}Zn_{0.7}Ti_{0.2}Zr_{0.8}Nb_2O_8$ supplemented with 20 mol% LiF

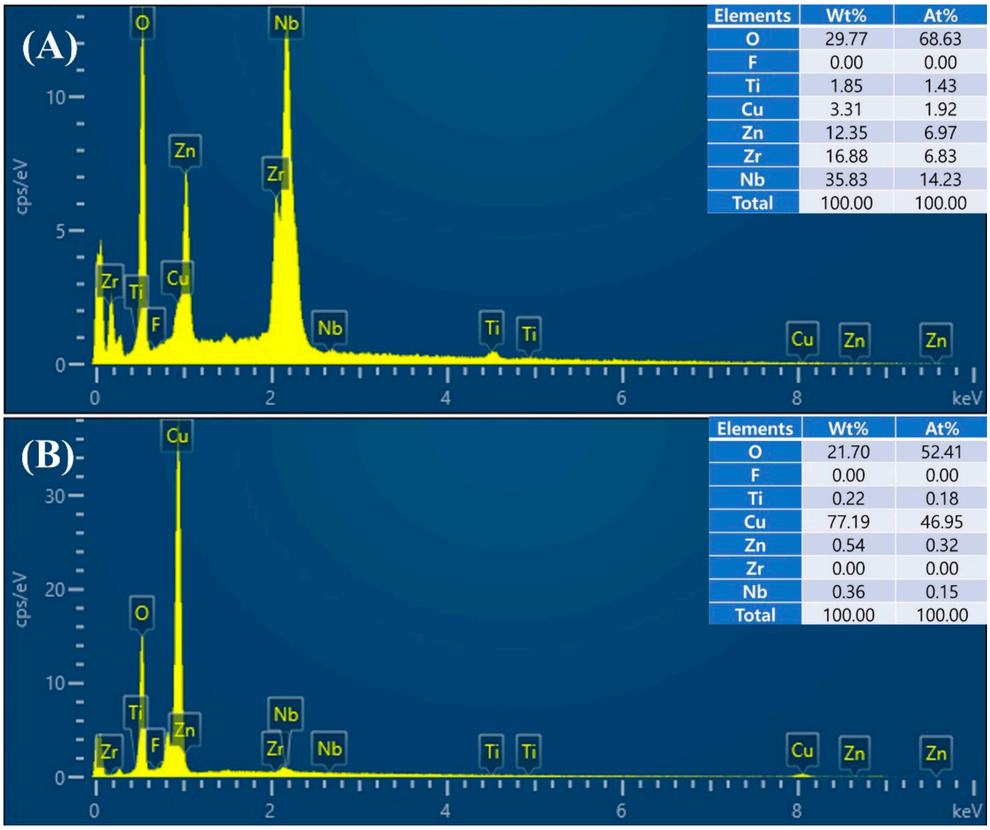

Figure S3. EDS analysis of $Cu_{0.3}Zn_{0.7}Ti_{0.2}Zr_{0.8}Nb_2O_8$ supplemented with 20 mol% LiF

5. EDS analysis of $Cu_{0.3}Zn_{0.7}Ti_{0.2}Zr_{0.8}Nb_2O_8$ supplemented with 40 mol% LiF with Ag cofired

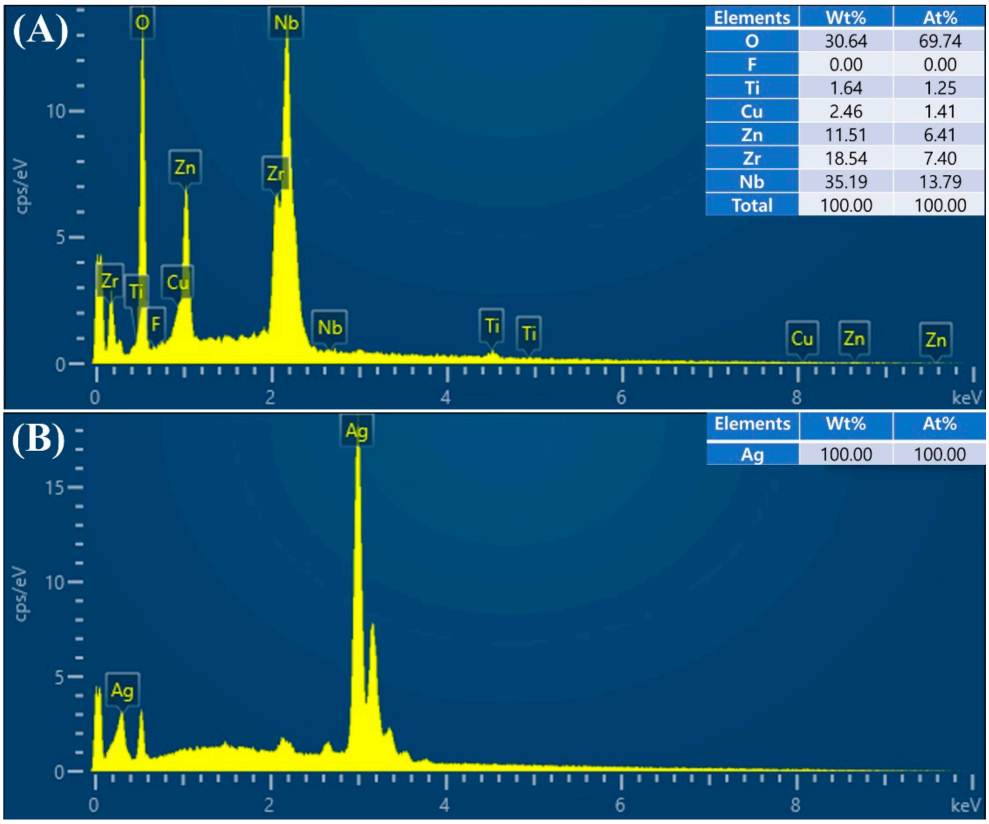

Figure S4. EDS analysis of $Cu_{0.3}Zn_{0.7}Ti_{0.2}Zr_{0.8}Nb_2O_8$ supplemented with 40 mol% LiF with Ag cofired
